# Use of a left ventricular assist device (Heart Mate III) as a destination therapy in a patient with chronic heart failure due to Chagas disease. Could it have been avoided?

**DOI:** 10.15446/rsap.V25n5.106562

**Published:** 2023-09-01

**Authors:** Tania M. Blanchar-Martínez, Fernando Pío De la Hoz-Restrepo, Seila R. López-Suárez

**Affiliations:** 1 TB: IQP. Epid. Grupo Investigación, Epidemiología y Evaluación en Salud Pública. Facultad de Medicina, Departamento en Salud Pública. Universidad Nacional de Colombia. Bogotá, Colombia. tmblanchar@gmail.com Universidad Nacional de Colombia Facultad de Medicina Departamento en Salud Pública Universidad Nacional de Colombia Bogotá Colombia; 2 FH: MD. M. Sc. Epidemiología. Ph. D. Salud Pública. Grupo Investigación, Epidemiología y Evaluación en Salud Pública. Universidad Nacional de Colombia. Bogotá, Colombia. fpdelahozr@unal.edu.co Universidad Nacional de Colombia Grupo Investigación, Epidemiología y Evaluación en Salud Pública Universidad Nacional de Colombia Bogotá Colombia fpdelahozr@unal.edu.co; 3 SL: Enf. Gerencia y Auditoría de Calidad en Salud. Facultad Ciencias de la Salud, Universidad Cooperativa de Colombia. Santa Marta, Colombia. seilals@hotmail.com Universidad Cooperativa de Colombia Gerencia y Auditoría de Calidad en Salud Facultad Ciencias de la Salud Universidad Cooperativa de Colombia Santa Marta Colombia

**Keywords:** Chagas, chronic, heart failure, medical device, trypanosoma cruzi, Colombia *(source: MeSH, NLM)*, Chagas, insuficiencia cardíaca crónica, dispositivo médico, trypanosoma cruzi, Colombia *(fuente: DeCS, BIREME)*

## Abstract

**Introduction:**

Chagas is an infectious disease that may produce chronic consequences such as cardiac failure (IC), enlarged esophagus and colon. Cardiac failure can shorten people's lives and severely affect their quality of life. There are not many alternatives to treat patients with advanced cardiac damage. Recently, it has been proposed that implantation of devices that provide mechanical circulatory support may help Chagas' patients with advanced IC to improve its quality of life.

**Objective:**

To analyze the case of a patient with Chagas disease who was not treated in a timely manner due to failures in primary care, the consequences on his health, and the cost of late treatment that could have been avoided.

**Materials and Methods:**

Diagnostic methods revealed damage at the functional and structural level of the heart, classifying the condition as CHF, which led to the implantation of a technological medical device (DMT).

**Results:**

We present here the results of implanting such a device (Heart Mate 3) in a 54-year-old Colombian male patient with a 2 years diagnosis of advanced (IC) as a consequence of chronic Chagas infection.

**Conclusion:**

Chagas disease represents one of the oldest communicable diseases in the region of the Americas. Public health programs today should redirect their prevention programs into predictive- type interventions to offer more timely treatment, reducing care expenses and positively impacting the health budget.

## RESUMEN

**Introducción:**

El mal de Chagas es una enfermedad infecciosa que puede tener consecuencias crónicas como insuficiencia cardiaca (IC) y agrandamiento del esófago o del colon. La IC puede acortar la vida de las personas y afectar severamente su calidad de vida. No existen muchas alternativas para tratar a los pacientes con daño cardiaco avanzado. Recientemente, se ha propuesto que la implantación de dispositivos que proporcionen soporte circulatorio mecánico puede ayudar a los pacientes de Chagas con IC avanzada a mejorar su calidad de vida.

**Objetivo:**

Analizar el caso de un paciente con enfermedad de Chagas no tratada de manera oportuna por fallas en la atención primaria, las consecuencias en su salud y el alto costo de un tratamiento tardío que pudo ser evitable.

**Materiales y Métodos:**

Los métodos diagnósticos evidenciaron el daño a nivel funcional y estructural del corazón, clasificando su afectación como una ICC, lo que llevó a la implantación del dispositivo médico tecnológico (DMT).

**Resultados:**

Presentamos los resultados de la implantación de un dispositivo de este tipo (Heart Mate 3) en un paciente masculino colombiano de 54 años con diagnóstico de IC avanzada (2 años antes) como consecuencia de infección chagásica crónica.

**Conclusión:**

La enfermedad de Chagas representa una de las enfermedades transmisibles más antiguas en la región de las Américas. Los programas de salud pública deben reorientar sus programas de prevención hacia intervenciones de tipo predictivo para ofrecer un tratamiento más oportuno, de manera que se reduzcan los gastos de atención y se impacte positivamente el presupuesto de salud.

**Figure 1:**
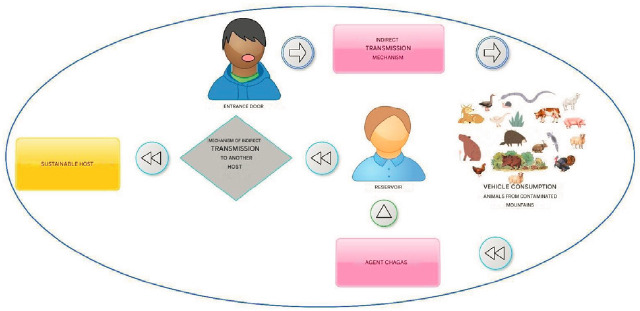
Chagas infection chain

Chagas is a disease with acute, subacute, and chronic phases caused by *Trypanosoma* cruzi (T. *cruzi*) infection 1. T. cruzi, is a pleomorphic hemoflagellate protozoan belonging to the class Mastigophora 2-4. It can be harbored by more than 100 animal species including wild and domestics animals. In Colombia, the main vector of T. Cruzi is a triatomine, *Rhodnius Prolixus,* but it could also be transmitted by blood transfusions (Figure 1).

In Colombia, Chagas infection is concentrated in the departments of Santander, Meta, Boyacá, and the Magdalena Valley.1 Chagas disease causes heart failure (AHF), which can be moderate, unilateral, acute, and chronic, depending on the involvement of the heart 5-7.

Primary strategies on Chagas disease prevention consist of eliminating the risk of infection and interrupting the chain of transmission through vector and non- vector control. Secondary prevention rests on screening and detection of individuals infected with T Cruzi, in the early stages of the disease, especially in acute cases. But for individuals in advanced stages of chronic diseases, tertiary prevention consists of clinical interventions to limit the morbidity and mortality of the disease and it may include surgical procedures 5. Recently, it has been proposed that implantation of devices that provide mechanical circulatory support may help Chagas' patients with advanced IC to improve its quality of life.

An exploratory search of the literature at a global and national level was carried out on the Hear mate 3 techno-logical device for the correction of chronic heart failure due to Chagas disease. The databases explored included Cochrane Library, LILACS, SciELO, MEDLINE, PubMed, and PubMed Central. We found no paper where a similar device had been used in a Chagas' patient.

Here, we present a case report on a patient with a chronic cardiopathy due to *Trypanosoma* cruzi infection who was implanted with Heart mate III, a new device providing mechanical circulatory support. This is likely the first of such reports in Colombia.

## MATERIALS AND METHODS

Original case study of a patient with Chagas disease. The methods used for the correct diagnosis of chronic heart failure caused by Chagas disease were based on serological tests for the detection of IgG antibodies, which detected the presence of T Cruzi. Additionally, the patient underwent different diagnostic tests that detected the presence of the disease due to functional and structural failures of the heart (Table 1).

**Table 1:** Differential diagnoses were found during care, before the implantation of the technological device (Hearmate III)

Diagnostic tests are performed before implantation of the technological device (Hearmate III)	
Type of examinations	Results Trans esophageal ultrasound, Doppler, trans thoracic echocardiogram, chest CT
Transesophageal ultrasound Doppler Transthoracic Echocardiography Chest CT scan	Steveson acute chronic heart failure. Chagasic dilated cardiomyopathy. Severely depressed LVEF 15% - 20%. Advanced biventricular Chagasic heart failure Severe pulmonary hypertension Moderate tricuspid and mitral insufficiency Atrial fibrillation and atrial flutter, unspecified Congestive heart failure. Cardiogenic Shock. Risk of right ventricular dysfunction CDI reprogramming.
Chest x-rays	Chest X-Ray Results
	Vascular pedicle without alteration Calcified atheromas on the aortic button. Prominence of the central pulmonary vasculature. Severe global cardiomegaly. A nodular image of a calcified appearance projected on the left lung base may correspond to residual nodules. Three-chamber synchronizing cardio. Right peripheral insertion catheter end in superior vena cava Risk of hemodynamic deterioration. Arterial hypertension.

## RESULTS

**Figure 2:**
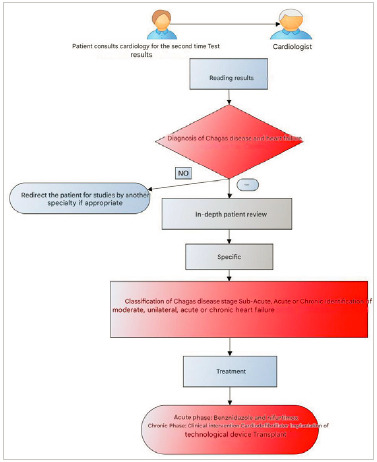
Diagnostic and treatment algorithm for Chagas disease and heart failure

Source: Own elaboration.

The patient was a 54-year-old male patient, of mixed race, who worked as a bus driver in a coal mine in the department of La Guajira, but was retired from work, with benefits, since 2017. In 2017, he was diagnosed with Chagas disease after consulting for symptoms compatible with chronic heart failure - exhaustion, fatigue, and difficulty breathing. (Figure 2).

**Figure 3:**
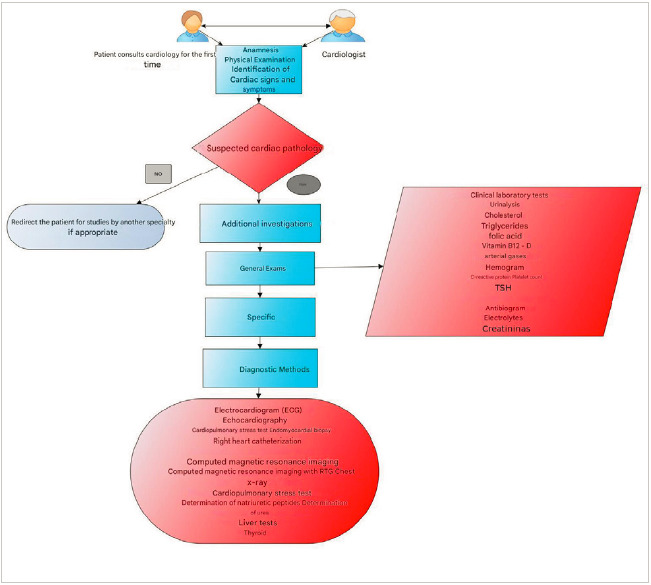
Algorithm of diagnostic methods for the evaluation of patients with suspected heart disease

Source: Own elaboration.

He was ordered a trans esophageal ultrasound doppler, a transthoracic echocardiography and a chest CT scan which reported a dilated cardiomyopathy, a severely depressed ejection fraction (15% - 20%), advanced biventricular heart failure, severe pulmonary hypertension, moderate tricuspid and mitral insufficiency, atrial fibrillation and atrial flutter. Chest X rays reported prominence of the central pulmonary vasculature and severe global cardiomegaly. (Figure 3).

An IgG test for T Cruzi infection yield positive so the patient was confirmed as a case of advanced cardiac failure with severe cardiac damage due to Chagas disease in 2017.

In 2022, after 5 years of clinical management and visible increase in the clinical and hemodynamic compromise, a joint medical board agreed that the best option to correct the chronic heart failure, and improve the quality of life of the patient, was through the implantation of a left ventricular mechanical device (Heart mate 3) using a minimally invasive heart surgery. He was considered not adequate for heart replacement because of a high pulmonary pressure.

**Figure 4:**
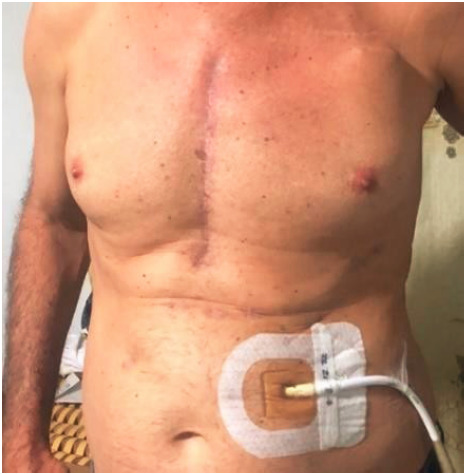
Sternotomy incision with technological device placement approach and lateral supraumbilical incision for the exit of the modular cable to connect to the mini-console

The surgical procedure consisted in a sternotomy incision to implant the left ventricular assist device -cardiac pump- at heart followed by a supraumbilical incision for the exit of the modular cable to the mini-console (Figure 4).

After 1 year follow up the patient has referred a substantial improvement in his quality of life and hemodynamic indicators.

## DISCUSSION

In this study, the use of HeartMate III for the correction of chronic heart failure in a patient with untreated Chagas disease was described. It was unknown how this patient was infected but he was born and lived his whole life in a semi-rural area in the department of La Guajira. This place has not been identified previously as an endemic area for Chagas disease, but has all the conditions to be one, such as presence of houses with mud walls, palm tree roofs, earthy floor, and close to rural areas with numerous palm trees where *Rhodnius prolixus* is known to live.

The patient spent almost 5 years waiting for a clinical intervention that could improve his quality of life and ameliorate his symptoms. He went from one health care institution to another, begging for a solution, undertaking which Abadia Barrero has called "bureaucratic itineraries" 8. Bureaucratic itineraries" are caused by an endless number of administrative barriers that health insurers have raised to control health expenditures but disproportionately affect poor and rural populations. In this case, the untimely intervention of the disease caused an important increase in suffering plus higher costs in medical care, because the time lag between date of diagnosis and date when a solution was implanted, and because the patient had to be transferred from his home town to Bogota, 600 miles away, to solve his clinical problem 9.

Among patients not eligible for heart transplantation, Heart Mate's patients had an important reduction of mortality (48%) compared to those under optimal medical management 10.

**Figure 5:**
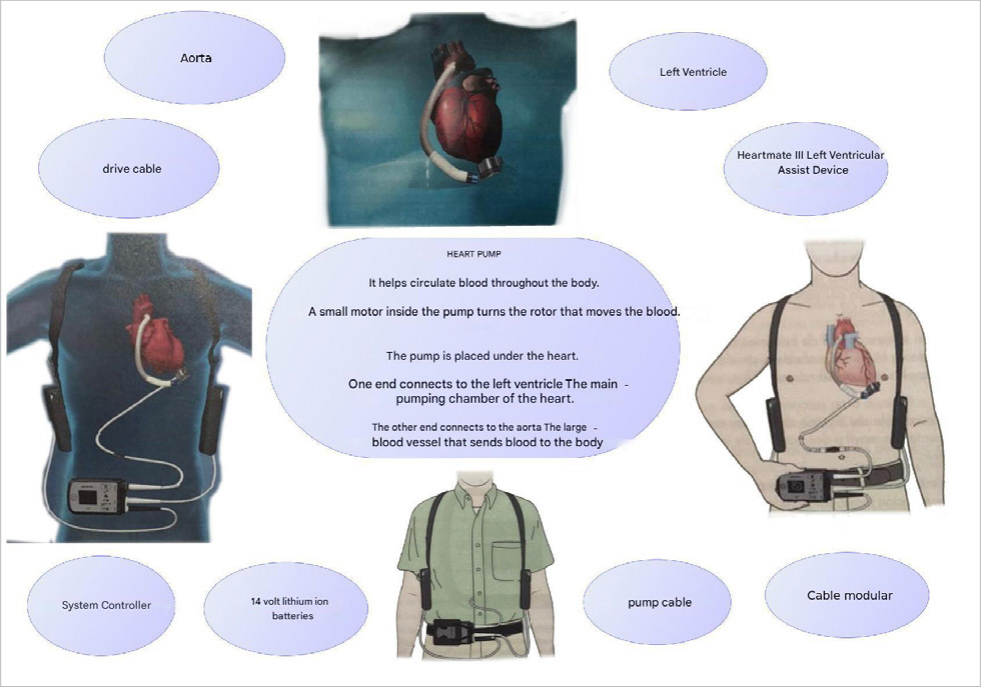
HeartMate III left ventricular assist

Source: taken and adapted from the Heartmate III Left Ventricular Device Patient Manual 12.

HeartMate is a left ventricular support system, able to generate a blood flow of up to 10 liters per minute (lpm). It connects to the apex of the left ventricle and to the ascending segment of aorta, diverts blood from the weakened left ventricle and propels it towards the rest of the body. It is controlled through an internal computer that regulates the pump (Figure 5).

The implantation of the device generated a change in the patient's quality of life since it allowed him to live a seemingly normal life despite the extensive damage to his circulatory system. However, there are still very few experiences in Latin America and worldwide with the use of this technology in Chagas patients. Most evidence comes from case series, with few patients, or case reports. The Brazilian Society of Cardiology (SBC) asserts that "currently, the major limitations to its applicability are high cost, RV dysfunction, and need for a specialized team for device implantation and management. The SBC guideline on mechanical circulatory support recommends careful assessment of the RV function as mandatory before implantation, and, in the presence of moderate to severe dysfunction, one should be prepared for biventricular support implantation" 11.
